# Physiological and autonomic responses to intermittent low‐lung‐volume apnoea and pain sensitivity: A randomized controlled pilot trial

**DOI:** 10.1113/EP093960

**Published:** 2026-09-04

**Authors:** Cristian Mendoza‐Arranz, Francisco De Asís‐Fernández, Guillermo Miravet‐Mingo, José Vicente León‐Hernández, José Fierro‐Marrero, Álvaro Reina‐Varona

**Affiliations:** ^1^ Physical Activity and Health Research Group (AFYS), Faculty of Health Sciences Rey Juan Carlos University Alcorcón Madrid Spain; ^2^ Department of Physical Therapy, Occupational Therapy, Rehabilitation and Physical Medicine Rey Juan Carlos University Alcorcón Madrid Spain; ^3^ Cognitive Neuroscience, Pain, and Rehabilitation Research Group (NECODOR), Faculty of Health Sciences Rey Juan Carlos University Alcorcón Madrid Spain; ^4^ Departamento de Fisioterapia Facultad de Ciencias de la Salud. Centro Superior de Estudios Universitarios La Salle Universidad Autónoma de Madrid Madrid Spain; ^5^ Motion in Brains Research Group, Centro Superior de Estudios Universitarios (CSEU) La Salle Universidad Autónoma de Madrid Madrid Spain; ^6^ Faculty of Medicine Health and Sports Department of Physiotherapy Universidad Europea de Madrid Villaviciosa de Odón Madrid Spain

**Keywords:** apnoea, autonomic nervous system, blood pressure, breath holding, cardiovascular physiological phenomena, hypoxia, pain threshold

## Abstract

Intermittent apnoeas may induce hypoalgesia; however, the effects of low‐lung‐volume static apnoea have not been investigated. Eighty healthy participants were randomized to an apnoea group (AG) or a control group (CG). The AG performed a 6‐min protocol of intermittent static apnoea at low lung volume (passive exhalation), whereas the CG breathed normally in the supine position. Pressure pain thresholds (PPTs) were assessed at the thumb, tibialis anterior and C7, together with heart rate (HR), oxygen saturation ($S_{pO_{2}}$), and systolic and diastolic blood pressure (SBP and DBP). Open‐ended questions explored sensations, emotions and adverse effects. ANCOVA (baseline pressure pain threshold) revealed no significant between‐group differences in PPT at thumb (mean difference [MD] = −0.209 kg/cm^2^; 95% CI: −0.584, 0.158; *P* = 0.268), tibialis (MD = 0.014 kg/cm^2^; 95% CI: −0.341, 0.354; *P* = 0.939), or C7 (MD = −0.276 kg/cm^2^; 95% CI: −0.669, 0.102; *P* = 0.169). Intrasession analyses showed higher values in the AG for $S_{pO_{2}}$ (MD = 0.598%, *P* = 0.009), percentage maximum heart rate (MD = 4.344%, *P* < 0.0001), SBP (MD = 15.344 mmHg, *P* < 0.0001) and DBP (MD = 14.273 mmHg, *P* < 0.0001). The AG reported more distress‐related sensations and emotions, whereas the CG described more comfort‐related experiences. Adverse effects were mild. A 6‐min intermittent low‐lung‐volume static apnoea did not induce hypoalgesia despite autonomic and cardiovascular activation and was well tolerated. These findings suggest respiratory‐induced autonomic stress may be insufficient to modulate nociceptive processing, highlighting the importance of physiological conditions under which breath‐hold interventions may influence pain.

Trial registration: This study was prospectively registered on 27 November 2023, at ClinicalTrials.gov (Identifier: NCT06158256).

## Abstract (highlights)

- **What is the central question of this study?**
  Does intermittent static low‐lung‐volume apnoea induce hypoalgesia, and how are cardiorespiratory and autonomic responses involved in this process in humans?
- **What is the main finding and its importance?**
  Intermittent static low‐lung‐volume apnoeas did not produce meaningful changes in pressure pain thresholds despite eliciting marked cardiorespiratory responses, including heart rate, oxygen saturation and blood pressure. These findings indicate a dissociation between autonomic cardiorespiratory responses and pressure pain sensitivity, and highlight the need for further research to determine the conditions under which respiratory‐induced autonomic stress influences nociceptive processing.

## 1 INTRODUCTION

Breath holding elicits the human diving response, a reflex pattern characterized by bradycardia, peripheral vasoconstriction with reduced blood flow to capillary beds, an increase in blood pressure (BP), spleen contraction, and an elevation in sympathetic activity, potentially mediated by stimulation of peripheral chemoreceptors (Foster & Sheel, 2005). Collectively, these mechanisms are thought to promote oxygen conservation and redistribution to vital organs (Andersson et al., 2002). When apnoea is performed at low lung volume, the physiological stress may be accentuated due to a reduced intrapulmonary oxygen reserve and greater mechanical load on the respiratory muscles, which can modify cardiovascular and autonomic responses compared with high‐volume conditions (Andersson & Schagatay, 1997).

Exercise‐induced hypoalgesia refers to a transient reduction in pain sensitivity following physical activity. This effect has been reported in healthy individuals across several exercise modalities but appears to be inconsistent in people with chronic musculoskeletal pain (Rice et al., 2019). Similar hypoalgesic responses have been suggested during static apnoea protocols (Jafari et al., 2016). Potential mechanisms include hypoxia and hypercapnia‐mediated modulation of central nociceptive processing, activation of arterial baroreceptors, and autonomic adjustments associated with the diving response, all of which may influence descending inhibitory pathways. However, the specific contribution of these physiological factors to apnoea‐related hypoalgesia remains unclear.

Lung volume appears to be a relevant determinant of the physiological response to apnoea. In professional breath‐hold divers, lower lung volumes have been associated with shorter apnoea duration, a more pronounced reduction in heart rate (HR), and lower BP at the end of breath holds (Andersson & Schagatay, 1997). These differences have been attributed to greater arterial chemoreceptor stimulation caused by hypoxia secondary to the diminished oxygen stores in the lungs, which may intensify diving bradycardia (Foster & Sheel, 2005). Whether these volume‐dependent responses are able to modulate pain perception differently from high‐volume apnoeas is still uncertain.

To date, one study has explored low‐lung‐volume apnoeas performed during walking and did not observe significant changes in pressure pain thresholds (PPTs) (Mendoza‐Arranz et al., 2024). Nevertheless, the hypoalgesic effect of low‐lung‐volume apnoea conducted in a static supine position, where postural, ventilatory and metabolic demands are minimized and the diving response can be examined under more controlled physiological conditions, has not yet been investigated. Consequently, the extent to which integrated respiratory and autonomic stress responses modulate nociceptive sensitivity remains unclear and whether postural conditions may influence the magnitude or expression of these responses is still unknown. Therefore, the primary aim of the present randomized controlled pilot trial was to assess the hypoalgesic effect of a 6‐min intermittent low‐lung‐volume apnoea intervention performed in a static position in healthy participants. Secondary objectives were to monitor HR and blood oxygen saturation ($S_{pO_{2}}$) during the intervention, and to evaluate BP responses before, during and after the protocol, as indicators of physiological tolerability. In addition, open‐ended questions regarding perceived sensations and emotions were included to explore the feasibility, acceptability and safety of the intervention from the participants’ perspective.

## 2 METHODS

A randomized controlled pilot study with two parallel arms was conducted between 2023 and 2024 in Madrid, Spain. The study was approved by the Ethics Committee of the Centro Superior de Estudios Universitarios La Salle (reference CSEULS‐PI‐016/2023) and was performed in accordance with the latest revision of *Declaration of Helsinki* in 2013. The trial was prospectively registered at ClinicalTrials.gov (NCT06158256; registered on 27 November 2023). The trial was designed and reported in accordance with the CONSORT guidelines, and the CONSORT checklist is provided as Supporting information at Zenodo, Appendix S1). A parallel‐group design was selected due to the potential risk of carryover effects associated with repeated apnoea exposure and the novelty of the intervention.

### 2.1 Participants

Participants were recruited through non‐probabilistic convenience sampling at the Centro Superior Universitario La Salle and CrossFit La Catedral, Madrid, Spain, located at an altitude of 657 m above sea level. Participants included students, staff, recreationally active adults and individuals from the surrounding community. Healthy subjects aged 18–64 were recruited, considering potential age‐related differences in pain sensitivity (El Tumi et al., 2017). An additional inclusion criterion was a resting $S_{pO_{2}}$ of ≥95%. Exclusion criteria were the presence of cardiovascular, respiratory, metabolic, neurological or musculoskeletal disorders (Sierra‐Silvestre et al., 2020); history of epilepsy (Guieu et al., 1992); pregnancy (Leźnicka et al., 2022); regular use of pharmacological treatments, including contraceptives, or drug use and recent alcohol consumption (Thompson et al., 2017), with tobacco use not considered an exclusion criterion and not assessed; pain on the day of assessment or frequent pain during the previous month; and engagement in moderate or vigorous physical activity within 24 h prior to testing (Zheng et al., 2021). All participants who met the eligibility criteria were included after providing written informed consent.

### 2.2 Randomization and blinding

Randomization was performed by an external researcher prior to participant enrolment using GraphPad Prism (GraphPad Software, Boston, MA, USA). Allocation followed a 1:1 ratio and was based on a simple randomization procedure. Only the intervention provider had access to the allocation sequence, and participants were unaware of their assignment until immediately before the intervention. The assessor responsible for measuring PPTs with the algometer remained blinded to group allocation throughout all assessments by leaving the room during the intervention.

### 2.3 Sample size

Considering the exploratory nature of the study and its focus on feasibility and safety, a total of 80 participants (40 per group) were recruited. No formal a priori power calculation was performed, and the sample size was based on methodological recommendations for pilot and exploratory studies involving continuous outcomes (Teare et al., 2014), which suggest that sample sizes in this range are appropriate to obtain reasonably stable estimates of variability for planning and interpretation purposes.

### 2.4 Interventions

Both groups completed a 6‐min intervention in the supine position on an examination table. Participants in the control group (CG) were instructed to breathe normally throughout the 6 min. In the apnoea group (AG), participants performed repeated low‐lung‐volume apnoeas consisting of a deep inhalation followed by a passive exhalation that did not reach the expiratory reserve volume, after which a 30‐s breath hold was initiated. Each apnoea was followed by 10 s of spontaneous breathing for recovery, and this 30:10‐s cycle was repeated continuously until the end of the 6‐min period. Timing was controlled using a mobile application, and the investigator provided standardized verbal cues to initiate apnoea and resume breathing. Before the intervention, participants received standardized verbal instructions and physically practised the low‐lung‐volume apnoea manoeuvre under investigator supervision until they were able to perform it correctly. During the intervention, participants were also continuously supervised to ensure that apnoeas were performed at low lung volume as instructed.

The 30:10‐s scheme was selected to induce transient physiological responses while maintaining safety and feasibility. Although these specific intervals have not been directly linked to changes in pain sensitivity, their use was informed by pilot testing and by previous research employing intermittent low‐lung‐volume breath holding and prolonged expiration to evoke autonomic adjustments without excessive discomfort or risk (Woorons et al., 2007). This protocol was considered achievable for healthy individuals and potentially adaptable to clinical populations with limited exercise tolerance.

### 2.5 Primary outcomes

PPT is defined as the minimum pressure required to evoke the first painful sensation (Fischer, 1987). Before baseline assessment, participants underwent a familiarization measurement using the algometer on the forearm. This procedure ensured that participants understood that they should indicate the point at which pressure first became painful before formal PPT measurements were performed. PPTs were assessed by a different blinded researcher at three anatomical sites on the right side: the C7 spinous process, the base of the distal phalanx of the thumb, and the belly of the anterior tibialis muscle. These locations were selected to evaluate pressure pain sensitivity across different body regions and to explore the potential systemic spread of a hypoalgesic response (Rolke et al., 2005). The order of assessment was randomized, with a 30‐s interval between consecutive measurements, in line with previous protocols (Ylinen et al., 2007).

Measurements were performed before and after the intervention using a digital algometer (PAIN TEST™ FPX 50, Wagner Instruments, Greenwich, CT, USA). The device has demonstrated high inter‐rater reliability for PPT assessment (Chesterton et al., 2007). To ensure consistency, measurement sites were marked prior to testing. Pressure was applied at a constant rate of 0.5 ± 0.1 kg/cm^2^/s, guided by a digital metronome to standardize the application across trials. This procedure has shown excellent test–retest reliability (Mailloux et al., 2021).

### 2.6 Secondary outcomes

Participants were characterized according to age, sex, body mass index (BMI), and previous experience with apnoea. Physical activity levels were assessed using the Global Physical Activity Questionnaire (GPAQ), which comprises 15 items on physical activity and one on sedentary behaviour, providing an estimate of weekly energy expenditure in metabolic equivalents (METs) (Bull et al., 2009). Perceived stress was evaluated with the 14‐item Perceived Stress Scale (PSS), scored on a five‐point Likert scale with total scores ranging from 0 to 56, where higher scores indicate greater stress (Remor, 2006). Sleep quality was assessed using the Pittsburgh Sleep Quality Index (PSQI), a 19‐item questionnaire, with total scores ranging from 0 to 21, in which higher scores reflect poorer sleep quality (Hita‐Contreras et al., 2014). These variables were included because they have been reported to influence pain thresholds (Årnes et al., 2023; Sivertsen et al., 2015; Timmers et al., 2018).

HR was recorded in the supine position at rest and throughout the intervention using a Polar H10 chest strap sensor (Polar Electro Oy, Kempele, Finland) (Schaffarczyk et al., 2022). The theoretical maximum heart rate (HRmax) was estimated using the equation: 208 − (0.7 × age) (Tanaka et al., 2001), and HR data were expressed as a percentage of HRmax (%HRmax) as a descriptive index of autonomic cardiovascular response. $S_{pO_{2}}$ was measured at rest and during the intervention with a finger pulse oximeter (NONIN Medical Inc., Model 9843; Plymouth, MN, USA) placed on the third finger of the right hand. BP was assessed in a seated position at rest and immediately after the intervention, and in the supine position at baseline before the intervention and subsequently every minute during the protocol (0, 1, 2, 3, 4 and 5), using an Omron Rs7 Intelli IT HEM‐6232T‐E wrist monitor (Omron Healthcare, Inc., Kyoto, Japan) (Tasic et al., 2019).

Perceived exertion was evaluated immediately after the intervention using the modified 10‐point Borg scale (Borg CR‐10) (Shariat et al., 2018). In addition, upon completion of the session, participants were asked open‐ended questions regarding the physical sensations and emotional responses experienced during the 6‐min protocol, as well as the occurrence of any adverse events.

### 2.7 Statistical analysis

All analyses were performed in R (v4.1.1; R Core Team, 2021). Details on the packages used and an expanded description of the statistical procedures are provided in Supporting information available at Zenodo (Methods S1). Sociodemographic characteristics and baseline measurements were described for each group using the mean and standard deviation (SD), the median with first (Q1) and third (Q3) quartiles, and the range for continuous variables, and frequencies for categorical variables. Data were displayed using density, scatter, Q–Q, and bar plots. Intrasession $S_{pO_{2}}$, %HRmax, systolic blood pressure (SBP), and diastolic blood pressure (DBP) values were also plotted over time and summarized using the same descriptive statistics.

Possible baseline differences between groups were examined with ordinary least squares (OLS) regression, and assumptions of homoscedasticity, linearity and normality were checked visually. When these three assumptions were considered acceptable, OLS models with 2000 bootstrap resamples (percentile) and a fixed seed were used to obtain robust estimates and 95% percentile confidence intervals (CIs) for the group mean difference (MD). When the previously described assumptions were not met, the Wilcoxon rank‐sum test (*W*) was used. Categorical variables were compared using the chi‐square test, or Fisher's exact test when expected cell counts were <5.

OLS regression was then used to analyse PPTs (C7, thumb and tibialis), Borg CR‐10, and seated SBP and DBP, with group as the primary predictor. Baseline PPTs, SBP and DBP were added as covariates within an analysis of covariance (ANCOVA) approach. Group comparisons between the AG and CG were expressed as mean differences with standard errors (SE) and bootstrap percentile 95% CIs.

To evaluate changes in $S_{pO_{2}}$, %HRmax, SBP and DBP during the intrasession period, generalized least squares (GLS) models were fitted using a continuous autoregressive correlation structure (CAR(1)) to account for within‐subject dependence over time. For $S_{pO_{2}}$ and %HRmax variables, each model adjusted for the baseline value and included Group (CG vs AG), Time (0–39 s, with 0 corresponding to the start of each 40‐s cycle), Intrasession cycle (1−9), and the Group × Time and Group × Time × Intrasession cycle interactions. Moreover, exclusively for the $S_{pO_{2}}$ analysis, the model was repeated excluding the first cycle due to aberrant behaviour during that cycle, which showed no change in saturation, likely attributable to a delay in the instrument's capture capacity. For SBP and DBP variables, each model adjusted for the baseline value and included Group (CG vs AG), Time (0–6 min) and the Group × Time interaction. Baseline values and time were modelled with restricted cubic splines (four knots at the 0.05, 0.35, 0.65 and 0.95 quantiles for $S_{pO_{2}}$ and %HRmax variables and three knots at the 0.1, 0.5 and 0.9 quantiles for SPB and DBP). Outcomes were reported as regression coefficients, including both the linear term (*b*) and the non‐linear spline components (*b*′ and *b″*), along with their SE, *t*‐statistics and *P*‐values. Model‐fitted values were plotted with 95% CIs.

## 3 RESULTS

### 3.1 Participants

**FIGURE 1:**
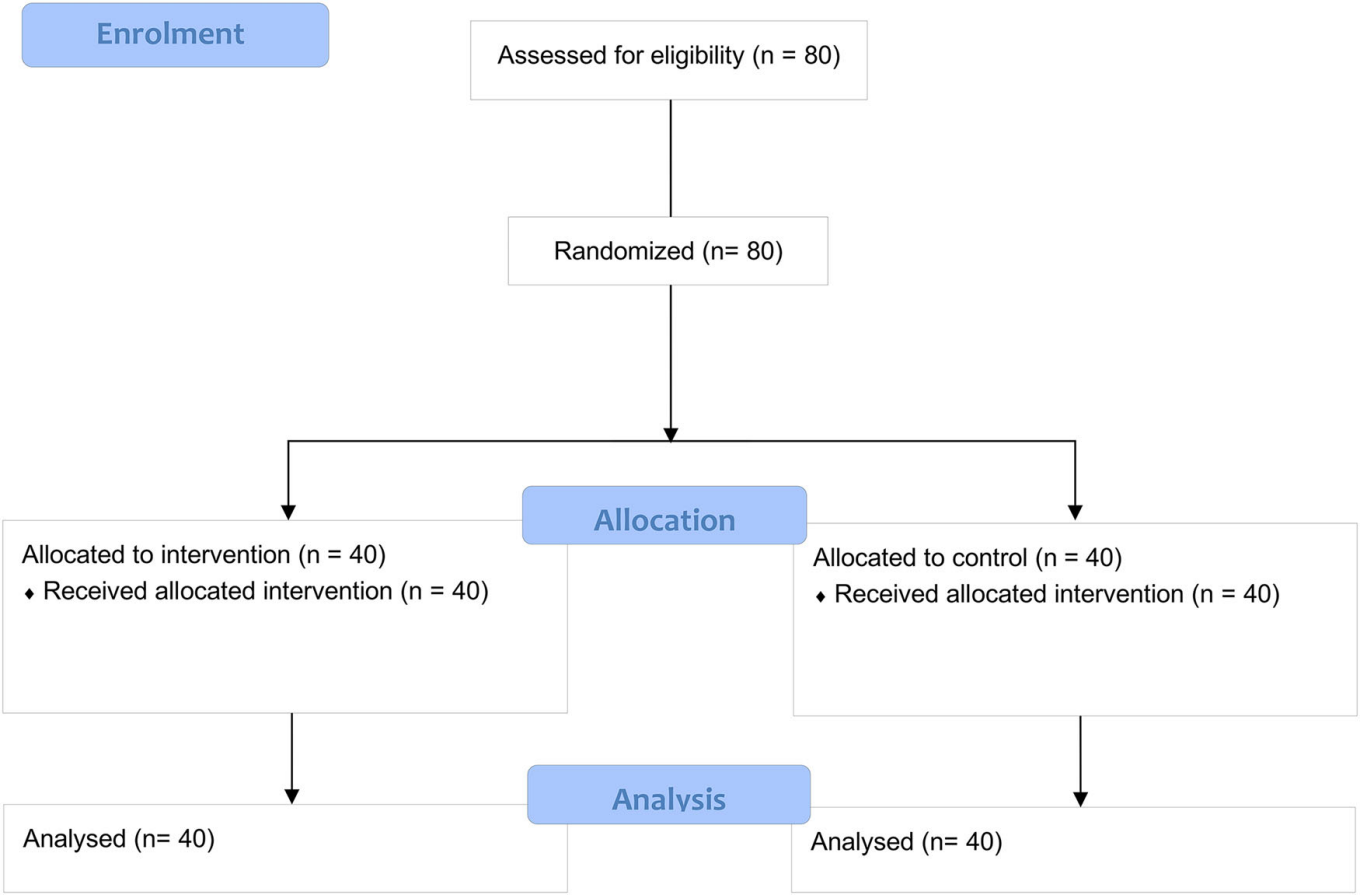
Participant flowchart following CONSORT recommendations.

A total of 80 participants were included in the study (Figure 1). Forty participants were allocated to the apnoea group (AG; 19 males and 21 females) and 40 to the control group (CG; 22 males and 18 females). All participants completed the intervention. Sociodemographic characteristics and baseline values are presented in Table 1, and graphical inspection of distributions (Q–Q plots, density plots and scatter plots) is provided in Supporting information at Zenodo (Results S1, Section 1). No relevant between‐group differences were identified in baseline variables. Detailed comparisons are reported in Supporting information at Zenodo (Results S2, Quantitative variables and Categorical variables).

**TABLE 1:** Baseline and sociodemographic characteristics and descriptives variables of both groups.

	Group AG: *n* = 40 CG: *n* = 40	Mean ± SD	Min value	Q1	Median	Q3	Max value	Between groups contrast
**Age (years)**	AG	24.7 ± 7.4	19	21	23	25.5	57	*W* = 766
	CG	25.8 ± 9.1	18	20	22	26.25	57	
**Sex (*n*, %)**	AG	M: 19, 47.5%; F: 21, 52.5%						χ^2^ = 0.2
	CG	M: 22, 55%; F: 18, 45%						
**BMI (kg/m^2^)**	AG	22.64 ± 3.7	16.5	20.7	21.9	24.1	35.2	*t* = −1.15
	CG	23.46 ± 2.6	20.3	21.5	22.9	24.9	30.8	
**GPAQ (MET)**	AG	4229.5 ± 3155.9	0	2025	3240	5790	12800	*W* = 808
	CG	4815.5 ± 5098.3	240	1910	3480	5820	28440	
**PSQI**	AG	5.6 ± 2.5	1	4	5.5	7.3	12	*t* = −0.99
	CG	6.25 ± 3.2	2	4	5.5	8	13	
**PSS**	AG	20.5 ± 7.3	4	16	22	25	35	*t* = −0.33
	CG	21.1 ± 8.7	5	15	20.5	28.3	37	
**Theoretical HR_max_ (bpm)**	AG	190.7 ± 5.4	167.1	190.1	191.9	193.4	194.9	*W* = 834
	CG	189.9 ± 6.7	167.1	189.6	192.7	194.1	195.6	
**Rest %HRmax**	AG	37.15 ± 5.15	26.4	33.2	37.5	40.7	47.6	*W* = 688
	CG	36.4 ± 8.2	24.6	30.6	36.3	40.9	61.8	
$S_{pO_{2}}$ **rest (%)**	AG	97.4 ± 1.2	95	96	98	98	99	*W* = 744
	CG	97.3 ± 1.1	95	97	98	98	99	
**SBP seated (mmHg)**	AG	123 ± 17.6	73	111.5	125	133	155	*W* = 860
	CG	125.3 ± 14	96	115.5	124.5	136.5	156	
**DBP seated (mmHg)**	AG	79.5 ± 12.6	42	72.25	80.5	86.5	103	*t* = −0.81
	CG	81.6 ± 11	62	72	80	91	104	
**SBP supine (mmHg)**	AG	122.9 ± 13.4	90	115.8	121	131.5	159	*W* = 781
	CG	122.9 ± 11.3	104	113	121	130.3	152	
**DBP supine (mmHg)**	AG	77.5 ± 11.4	45	70	78	86	108	*t* = 0.00
	CG	77.5 ± 8.9	63	71.5	77	82.5	104	
**PPT thumb (kg/cm^2^)**	AG	4.38 ± 1.95	1.19	2.82	4.03	5.46	10.36	*t* = 1.42
	CG	3.82 ± 1.59	1.43	2.95	3.62	4.56	9.74	
**PPT tibialis (kg/cm^2^)**	AG	5.64 ± 2.41	1.70	3.88	5.16	6.82	12.73	*t* = 0.78
	CG	5.25 ± 2.06	1.31	3.73	5.69	6.25	10.3	
**PPT C7 (kg/cm^2^)**	AG	3.86 ± 1.67	0.61	2.81	3.77	4.69	8.96	*t* = 0.37
	CG	3.73 ± 1.52	1.14	2.62	3.73	4.40	7.77	

*Note*: There were no between group differences. Abbreviations: AG, apnoea group; BMI, body mass index; bpm, beats per minute; CG, control group; DBP, diastolic blood pressure; F, female; GPAQ, Global Physical Activity Questionnaire; HRmax, maximum heart rate; MET, metabolic equivalent of task; PPT, pressure pain threshold; PSQI, Pittsburgh Sleep Quality Index; PSS, Perceived Stress Scale; Q1, quartile 1; Q3, quartile 3; SBP, systolic blood pressure; SD, standard deviation; $S_{pO_{2}}$, oxygen saturation; *t*, *t*‐value; *W*, Wilcoxon's rank‐sum test; χ^2^, chi‐square statistic.

### 3.2 PPTs

OLS model assumptions and diagnostic plots for the three PPT locations (thumb, tibialis, and C7) are provided in Supporting information available at Zenodo (Results S1, Section 2). Q–Q plots indicated that residuals followed the theoretical quantiles, supporting normality. Plots of residuals against fitted and baseline values showed approximately constant variance within the range of −2 to 2, supporting homoscedasticity. Inspection of residuals versus fitted and baseline values revealed no systematic patterns, supporting the linearity assumption. Overall, model assumptions were considered to be adequately met for all three ANCOVA models. Post‐intervention values for the three regions are presented in Table 2.

**TABLE 2:** Post‐intervention outcomes in both groups.

	Group AG: *n* = 40 CG: *n* = 40	Mean ± SD	Min value	Q1	Median	Q3	Max value
**PPT thumb post (kg/cm^2^)**	AG	4.33 ± 2.02	1.31	2.81	3.81	5.52	9.88
	CG	4 ± 1.78	1.03	3.12	3.66	5.06	9.39
**PPT tibialis post (kg/cm^2^)**	AG	5.47 ± 2.43	1.79	3.73	5.07	6.74	12.79
	CG	5.08 ± 2.19	1.03	3.48	4.95	6.43	9.88
**PPT C7 post (kg/cm^2^)**	AG	3.86 ± 1.77	0.75	2.68	3.64	4.54	8.24
	CG	4 ± 2.05	0.87	2.51	3.59	5.23	10.86
**SBP seated post (mmHg)**	AG	122.4 ± 15.61	80	116.5	123	133.25	149
	CG	122.23 ± 12.48	90	116.5	122	130	148
**DBP seated post (mmHg)**	AG	79.4 ± 11.45	49	70	82	88	96
	CG	78.45 ± 10.56	53	73.75	78	85.25	101
**Borg CR‐10**	AG	4.45 ± 1.99	0	3	4.5	6	8
	CG	0.1 ± 0.36	0	0	0	0	2

Abbreviations: AG, apnoea group; CG, control group; DBP, diastolic blood pressure; PPT, pressure pain threshold; Q1, quartile 1; Q3, quartile 3; SBP, systolic blood pressure; SD, standard deviation

#### 3.2.1 Thumb PPTs

For the thumb site, the OLS model yielded an *R* ^2^ of 0.804 and an adjusted *R* ^2^ of 0.799. The ANCOVA analysis showed no significant difference in PPT between groups (*F*(1, 77) = 1.25, *P* = 0.268). The estimated contrast between the AG and CG indicated a mean difference (MD) of −0.209 kg/cm^2^ (SE = 0.187; 95% CI: −0.584, 0.158).

#### 3.2.2 Tibialis PPTs

For the tibialis anterior, the OLS model showed an *R* ^2^ of 0.880 and an adjusted *R* ^2^ of 0.877. No significant between‐group difference was observed (*F*(1, 77) = 0.01, *P* = 0.939). The estimated contrast yielded an MD of 0.014 kg/cm^2^ (SE = 0.176; 95% CI: −0.341, 0.354).

#### 3.2.3 C7 PPTs

For the C7 site, the OLS model produced an *R* ^2^ of 0.793 and an adjusted *R* ^2^ of 0.788. The ANCOVA model did not show a significant difference between groups (*F*(1, 77) = 1.92, *P* = 0.169). The estimated contrast showed an MD of −0.276 kg/cm^2^ (SE = 0.199; 95% CI: −0.669, 0.102).

**FIGURE 2:**
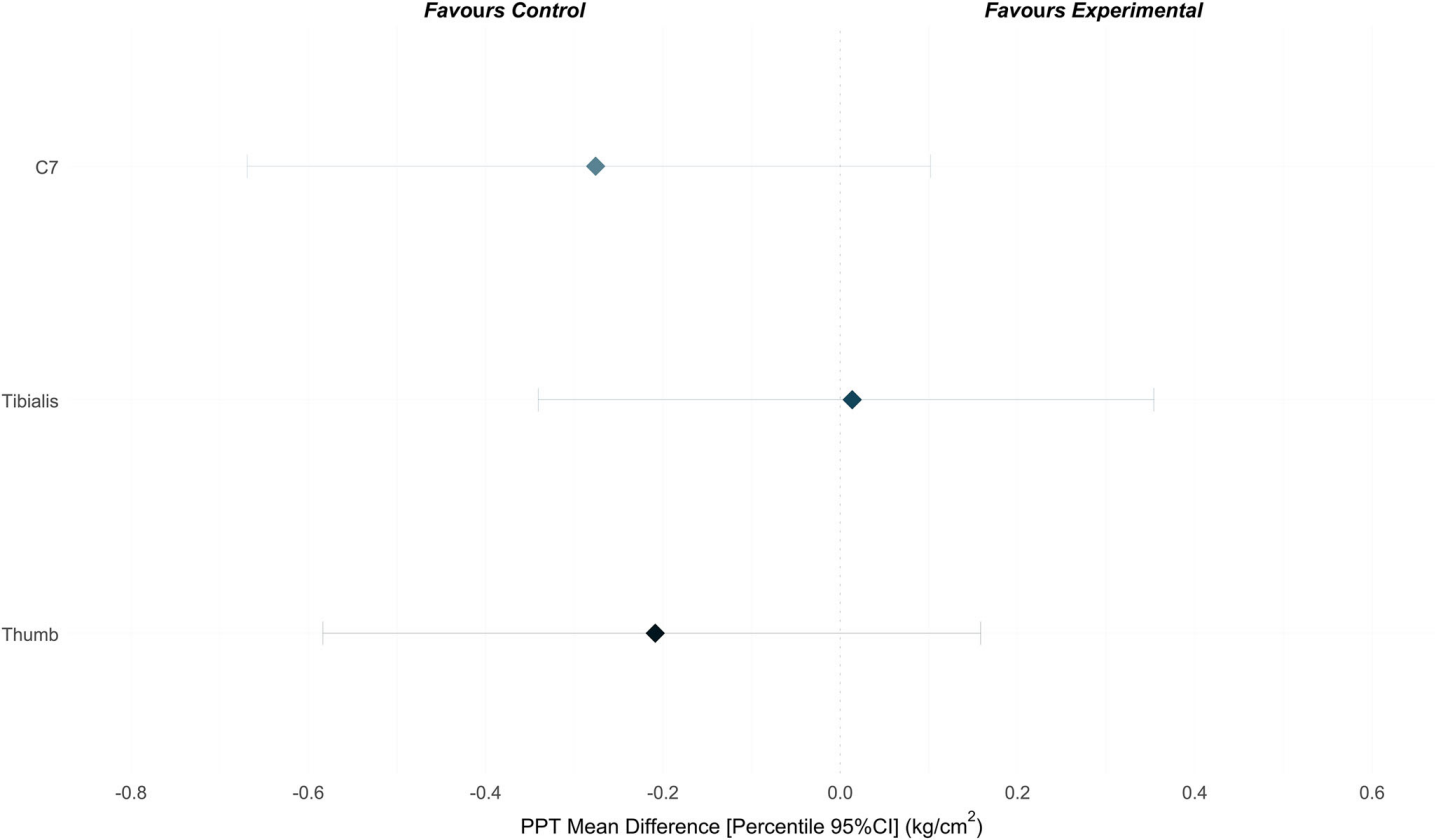
PPT means difference and 95% CI of thumb, tibialis and C7. The figure indicates the estimated mean differences between groups, and 95% CI mean difference values based on percentile method.

Mean differences with their corresponding 95% CIs for all three sites are shown in Figure 2.

### 3.3 Oxygen saturation and heart response during intervention

Model assumptions of normality and homoscedasticity were generally satisfied for both physiological outcomes. For $S_{pO_{2}}$, Q–Q and residual plots suggested mild deviations from normality and a potential heteroscedastic pattern; however, given the sample size, departures from normality were considered unlikely to substantially affect the validity of the analyses (Supporting information at Zenodo; Results S1, Sections 3.1−3.4 and 4).

**FIGURE 3:**
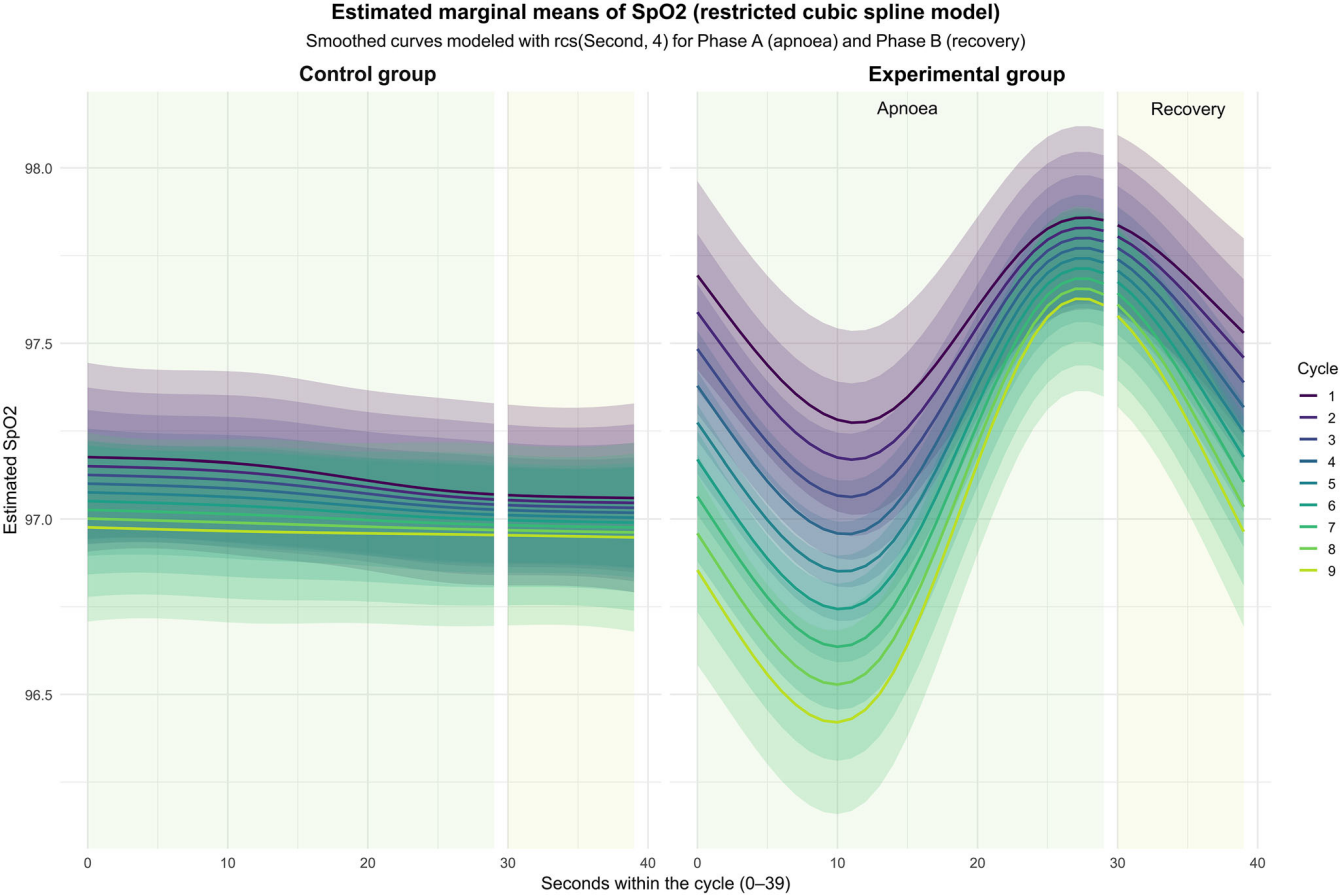
Intrasession oxygen saturation ($S_{pO_{2}}$) predicted values across the intervention period. Curves represent model‐predicted $S_{pO_{2}}$ values over the intervention time (0–360 s) across all apnoea cycles (1–9), adjusted for baseline resting $S_{pO_{2}}$. The first cycle was included in this analysis.

When the analysis included the first cycle, a significant between‐group difference in $S_{pO_{2}}$ was observed at the reference time point (MD = 0.598%; SE = 0.229; 95% CI: 0.149, 1.048; *t* = 2.61; *P* = 0.009), with slightly higher values in the AG compared with the CG. No significant main effects of time (Time, *t* = −0.08; Time′: *t* = −0.35; and Time″, *t* = 0.38; *P* > 0.05 for all) or cycle were detected. However, a significant Group × Time interaction (*P* < 0.0014 for all) indicated different temporal trajectories between groups. The AG showed an initial decrease during the first seconds of apnoea, followed by a gradual increase towards the end of the apnoea period, and a subsequent decrease. In contrast, the CG displayed a relatively stable profile across the same period. This pattern was further influenced by cycle number, as reflected by a significant Group × Cycle × Time interaction, with late‐phase desaturation becoming progressively more evident across successive cycles in the AG (Figure 3).

After excluding the first cycle, the overall pattern of results remained consistent. Detailed model results, and the full description of the analysis are reported in Supporting information at Zenodo (Results S1, Section 3.5, and Results S2, GLS Intrasession SpO_2_ and GLS Intra no 1st cycle SpO_2_).

**FIGURE 4:**
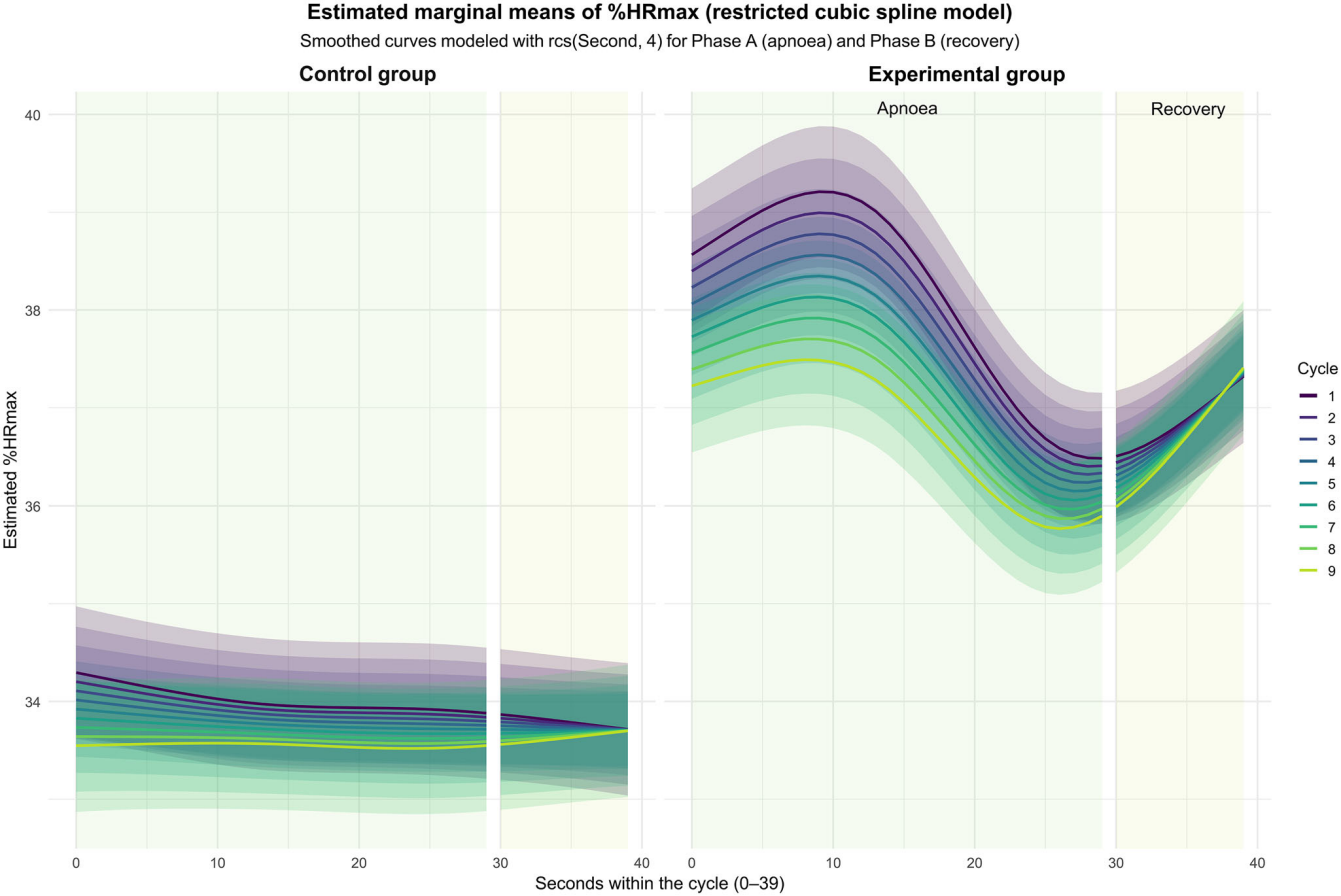
Intrasession percentage of maximal heart rate (%HRmax) predicted values across the intervention period. Curves represent model‐predicted %HRmax values over the intervention time (0–360 s) across all apnoea cycles (1–9), adjusted for baseline theoretical HRmax.

Regarding HR, the AG exhibited higher %HRmax than the CG at the reference time point (MD = 4.344%; SE = 0.578; 95% CI: 3.21, 5.477; *t* = 7.51; *P* < 0.0001). A significant main effect of time (Time, *t* = −2.08, *P* = 0.0374; Time′, *t* = 1.29, *P* = 0.198; Time″, *t* = −1.24, *P* = 0.214) indicated overall changes in %HRmax throughout the intervention. In addition, a significant Group × Time interaction (*P* < 0.0001) revealed different temporal patterns between groups. The AG showed an initial rise in %HRmax, followed by a transient reduction and a subsequent but less pronounced increase toward the end of the protocol, whereas the CG maintained a more stable trajectory during the same period. This response was further modulated by cycle number, as demonstrated by a significant Group × Cycle × Time interaction, with the influence of successive cycles being attenuated early in the session, amplified during the mid‐phase, and reduced toward the end (Figure 4). Complete model results are provided in Supporting information at Zenodo (Results S2, GLS Intrasession %HRmax).

### 3.4 Blood pressure during intervention

Model assumptions were generally satisfied for intrasession SBP and DBP (Supporting information at Zenodo, Methods S1, Section 5). During the intervention in the supine position, both SBP and DBP were higher in the AG compared with the CG (SBP: MD = 15.344 mmHg; SE = 2.307; 95% CI: 10.823, 19.866; *t* = 6.65; *P* < 0.0001; DBP: MD = 14.272 mmHg; SE = 1.688; 95% CI: 10.963, 17.581; *t* = 8.45; *P* < 0.0001). Overall, BP values showed relatively stable profiles over time.

**FIGURE 5:**
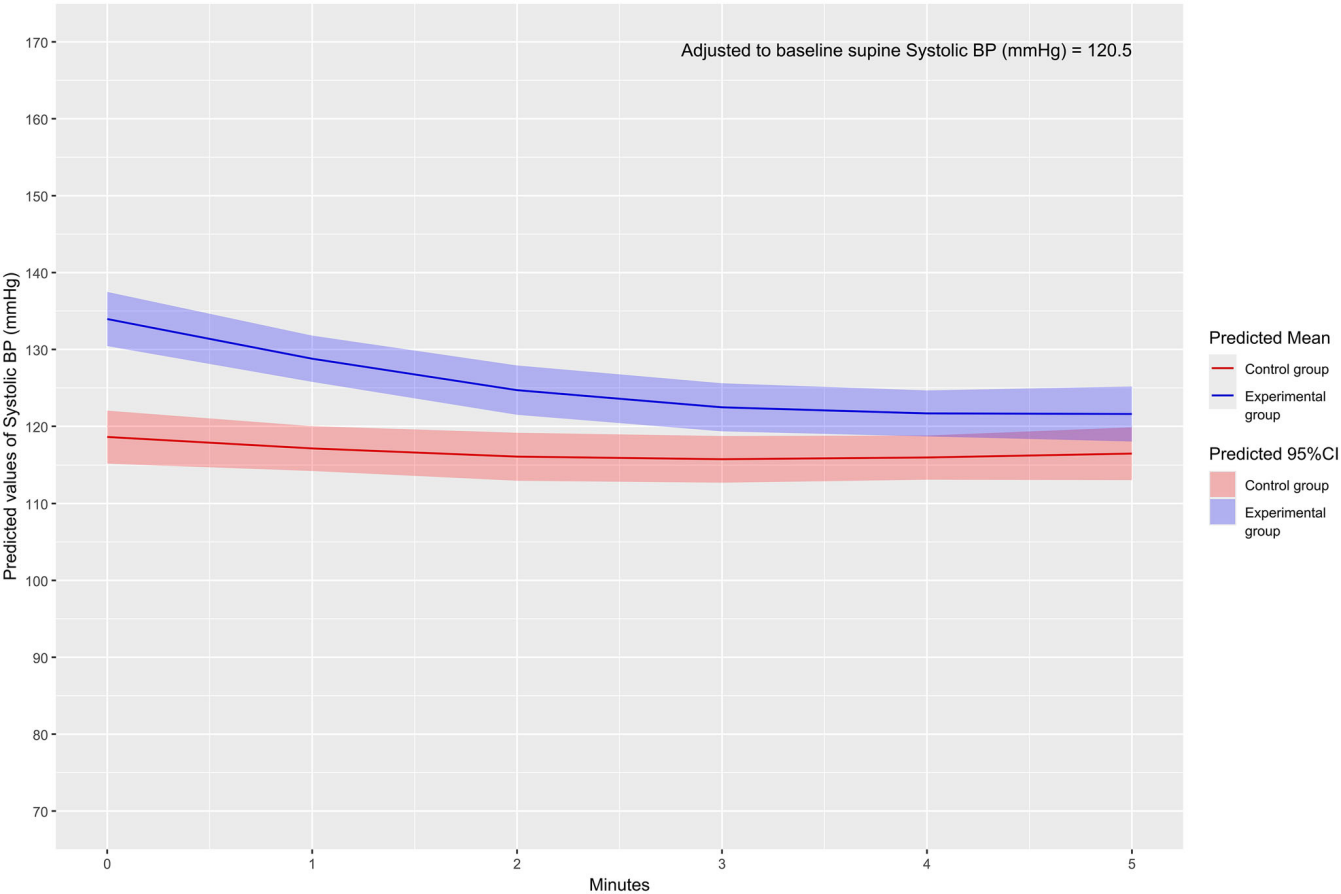
Intrasession systolic blood pressure (SBP) predicted values across the intervention. Curves represent model‐predicted SBP values measured at baseline (0 min) and at minutes 1, 2, 3, 4, 5 adjusted for baseline resting supine SBP.

**FIGURE 6:**
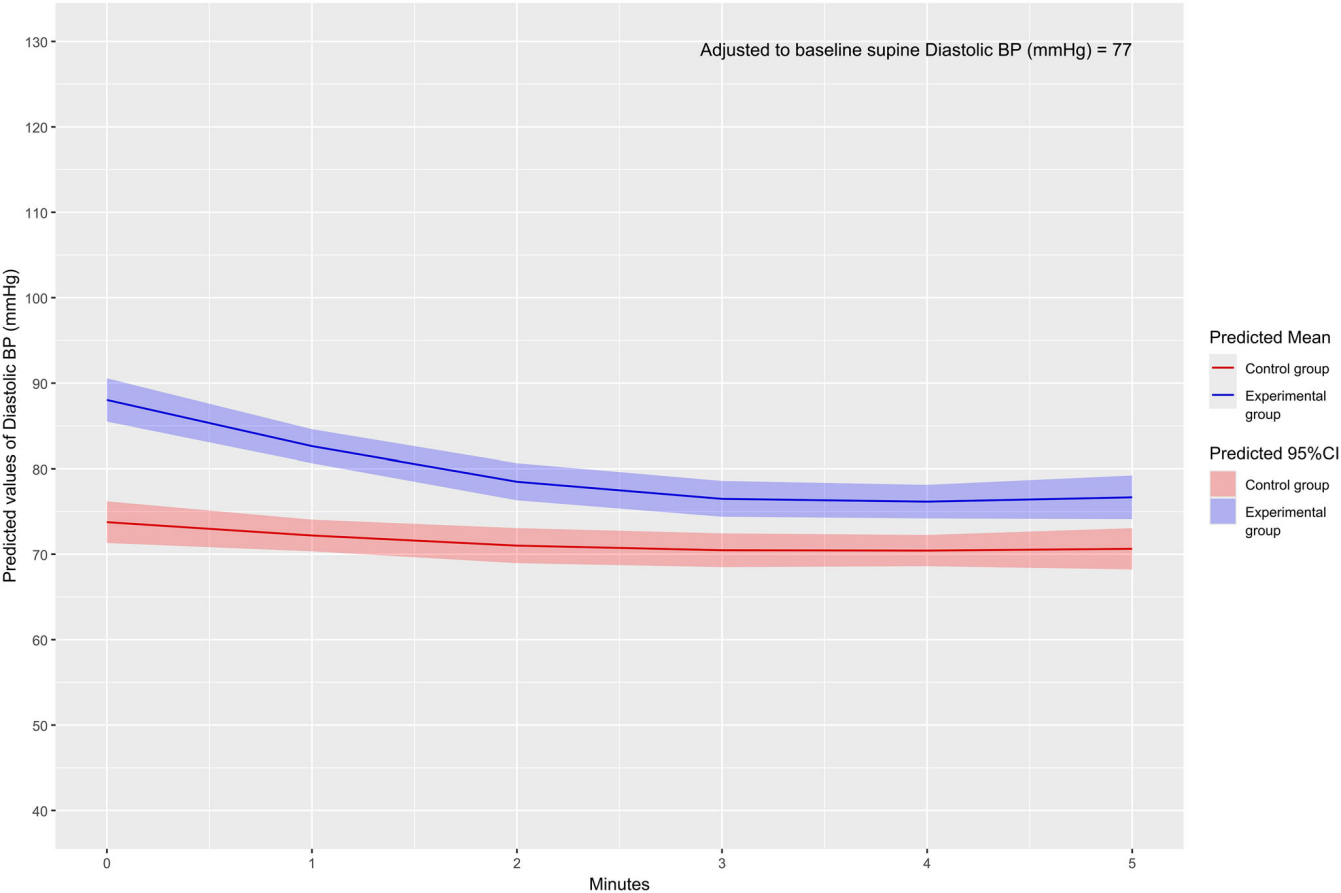
Intrasession diastolic blood pressure (DBP) predicted values across the intervention. Curves represent model‐predicted DBP values measured at baseline (0 min) and at minutes 1, 2, 3, 4, 5 adjusted for baseline resting supine DBP.

A significant Group × Time interaction was identified for SBP (*b* = −3.788, *P* = 0.011), indicating different temporal trajectories between groups, with the AG exhibiting a progressive reduction across the session and no evidence of a group‐specific curvilinear component. Similarly, a significant Group × Time interaction was observed for DBP (*b* = −3.985, *P* = 0.001), with the AG showing a steeper initial decrease over time and a significant positive curvilinear term (Group × Time′ = 3.65, *P* = 0.040) compared with the CG (Figures 5 and 6). Detailed model outputs are provided in Supporting information at Zenodo (Results S2, GLS Intrasession SBP and DBP).

### 3.5 Blood pressure after the intervention and perceived exertion

Model assumptions were generally met for seated SBP and DBP, whereas assumptions were not fully satisfied for perceived exertion (Supporting information at Zenodo, Methods S1, Section 6). Post‐intervention results for these variables are presented in Table 2. Post‐intervention seated measures showed modest explanatory capacity (SBP: *R* ^2^ = 0.302, adjusted *R* ^2^ = 0.284; DBP: *R* ^2^ = 0.291, adjusted *R* ^2^ = 0.272), with no relevant between‐group differences after the intervention (SBP: MD = 1.26 mmHg; SE = 2.659; 95% CI: −4.30, 6.082; *t* = 0.47; *P* = 0.637; DBP: MD = 2.001 mmHg; SE = 2.061; 95% CI: −2.111, 5.993; *t* = 0.97; *P* = 0.335).

Perceived exertion assessed with the Borg CR‐10 scale was significantly higher in the AG than in the CG (*W* = 27, *P* < 0.001). Reported scores ranged from 0 to 8 in the AG and from 0 to 2 in the CG.

### 3.6 Sensations, emotions and adverse effects

**FIGURE 7:**
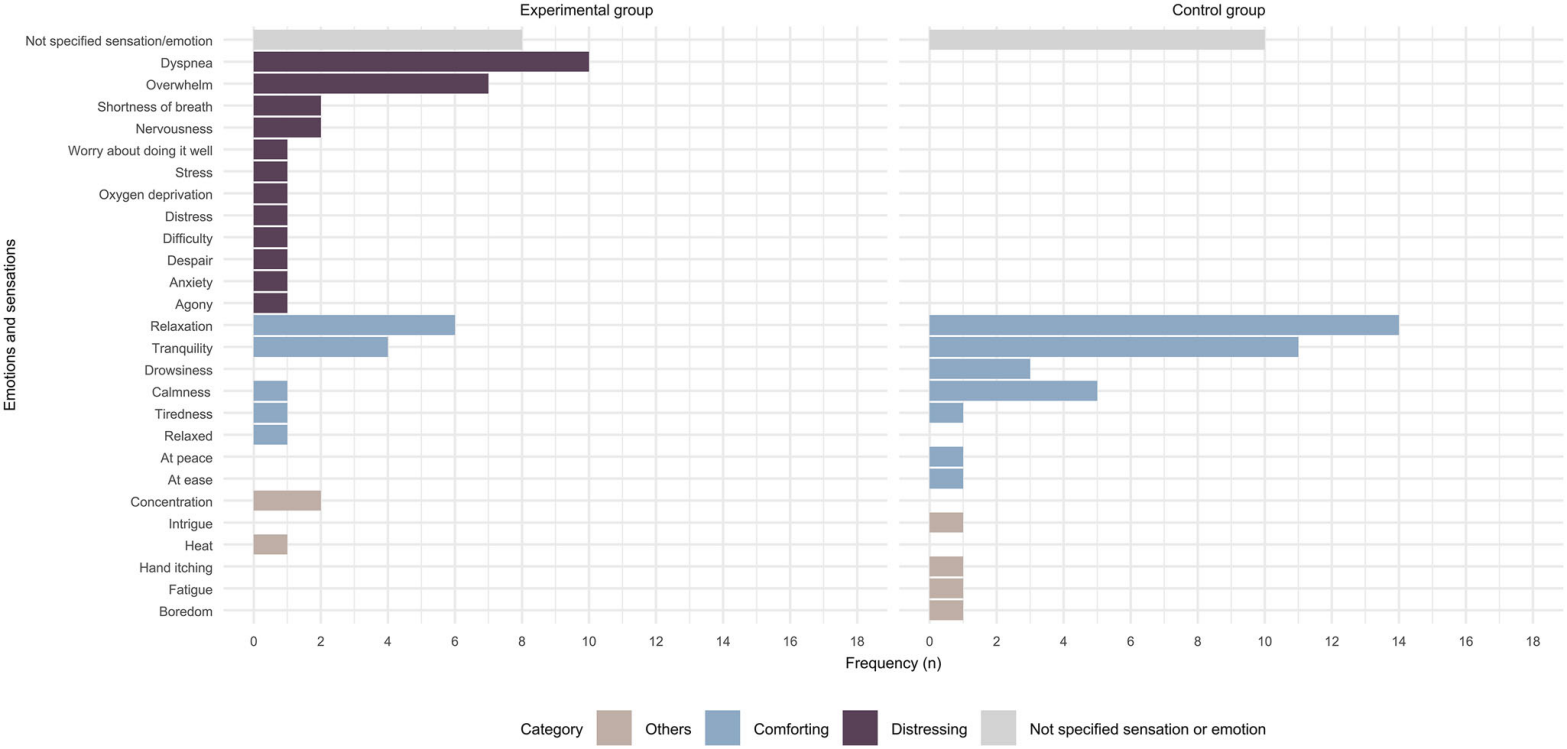
Emotions and sensations bar plot. The figure displays the frequencies of reported emotions and sensations by participants in each group. Additionally, emotions and sensations were categorized as comforting, distressing, others, and not perceived sensation or emotion classification.

Self‐reported sensations and emotions were categorized into four groups (comforting, distressing, not perceived sensation/emotion, and others) and are summarized in Figure 7. A higher proportion of participants in the AG reported distressing sensations and emotions (dyspnoea and overwhelm), whereas the CG more frequently reported comforting experiences (relaxation, tranquillity and calmness).

With respect to adverse effects, symptoms reported immediately after the intervention were infrequent and mild. In the AG, one participant reported mild dizziness, while in the CG one participant reported headache.

## 4 DISCUSSION

This pilot study investigated the integrative physiological and autonomic responses to intermittent low‐lung‐volume apnoea and their potential association with pain sensitivity in humas. PPT values at the thumb and C7 showed small between‐group differences, whereas measurements at the tibialis anterior were almost equivalent between groups. Confidence intervals for the thumb and C7 were wide, limiting interpretability, while the tibialis anterior CI was narrower and centred near zero, indicating no meaningful difference. Overall, not clinically meaningful between‐group differences in PPT were observed under the conditions of the present study.

A distinctive aspect of this study was the use of a 6‐min low‐lung‐volume apnoea protocol, which has not been previously examined in a static supine context. The absence of between‐group differences in PPT, consistent with finding from other studies using similar lung volumes (Mendoza‐Arranz et al., 2024), may be related to reduced baroreceptor stimulation at low‐lung volumes (Venezia et al., 2024), potentially due to smaller BP elevations compared with high‐lung‐volume apnoeas (Andersson & Schagatay, 1997). Although low‐lung‐volume apnoea is associated with greater hypoxic and hypercapnic stress, conditions that have been linked to pain modulation (Guekos et al., 2025), this could have promoted earlier and more pronounced chemoreflex activation (Foster & Sheel, 2005). We hypothesize that such a response could increase sympathetic drive and respiratory discomfort, thereby counteracting baroreceptor‐mediated or centrally driven hypoalgesic mechanisms.

$S_{pO_{2}}$ differed between groups irrespective of first‐cycle inclusion, with a progressive decline across cycles in the AG. This pattern may reflect a rightward shift of the oxyhaemoglobin dissociation curve. Repeated apnoeas favour CO_2_ accumulation and a reduction in blood pH, which enhance haemoglobin–oxygen dissociation and facilitate peripheral oxygen unloading, leading to greater hypoxaemia (Hamilton et al., 2004). Additionally, the intermittent structure of the protocol may contribute to cumulative desaturation: gas exchange is absent during apnoea, while the short recovery periods rely on limited oxygen uptake and CO_2_ clearance. As cycles progress, increasing metabolic demand and insufficient recovery could prevent full restoration of oxygen stores.

The higher initial HR observed in the AG may be explained by an anticipatory cardiorespiratory response described prior to exertional tasks (Miyamoto et al., 2022). The more pronounced mid‐cycle HR decrease in the AG is consistent with apnoea‐induced bradycardia (Hansel et al., 2009) and may be reinforced by residual hypoxaemia reflected in lower $S_{pO_{2}}$. Despite preserved or increased cardiac output, reduced oxygen availability could activate oxygen‐conservation mechanisms (Andersson et al., 2002) involving enhanced parasympathetic activity. Conversely, the late‐phase HR increase during recovery may be related to augmented respiratory frequency. The attenuated bradycardic response in later cycles may indicate that HR approached its physiological lower limit.

Intrasession SBP and DBP were initially higher in the AG and declined throughout the session. These early elevations may be associated with anticipatory stress and emotional arousal (Gordon & Mendes, 2021), amplified by the unfamiliar and potentially distressing nature of breath holding. Sympathetic activation during the initial apnoeas may transiently increase cardiac output and BP. Progressive familiarization with the task may reduce nervousness, contributing to subsequent decreases in HR and BP (Hoffmann et al., 2005).

Peak supine BP values recorded in the AG (SBP 178 mmHg, DBP 135 mmHg) were within or close to established ACSM exercise safety limits (approximately 220 mmHg for SBP and 105 mmHg for DBP) (Pescatello et al., 2004). The absence of post‐intervention differences in seated BP suggests that the protocol did not induce residual hemodynamic effects, consistent with previous observations (Hoffmann et al., 2005).

Higher Borg CR‐10 scores in the AG, together with a broader range of responses, may be related to apnoea‐induced hypercapnia, which can increase anxiety perception (Harrison et al., 2022) and amplify perceived exertion relative to normal breathing. Participants in the AG more frequently reported unpleasant sensations, whereas the CG described predominantly comfortable experiences. This pattern aligns with the greater perceived intensity typically associated with apnoea.

Several limitations should be acknowledged. First, the modest sample size, although appropriate for a pilot design, limits statistical power and precision. Additionally, although baseline differences were accounted for using ANCOVA, a within‐subject design could have further reduced inter‐subject variability. Second, the predominance of young healthy adults could restrict generalizability to older individuals or clinical populations with pain. Third, in the intrasession BP analysis, the intrasession cycle (apnoea and breathing phase) could not be incorporated due to variable measurement timing across participants, warranting cautious interpretation. Finally, apnoea performance was not objectively monitored; therefore, although all apnoeas were performed at low lung volume following standardized instructions, exact ventilatory volumes and individual differences in breathing strategy could not be quantified or fully controlled, and future studies should incorporate gas analysis to enhance protocol fidelity and enable more precise characterisation of ventilatory parameters.

This trial represents the first investigation of static low‐lung‐volume apnoea with an integrative physiological framework examining cardiorespiratory and autonomic responses alongside pain sensitivity in humans. Breath‐holding is increasingly used as a model of respiratory‐induced systemic stress, eliciting coordinated adjustments in autonomic and cardiovascular function (Wein et al., 2007). High‐lung‐volume apnoeas have been associated with baroreflex‐mediated hypoalgesia (Jafari et al., 2016), but the present findings showed no meaningful between‐group differences in PPTs following intermittent low‐lung‐volume static apnoea in healthy adults. These findings are consistent with the possibility that lung volume may influence the balance between baroreflex and chemoreflex activation, thereby influencing both cardiovascular and pain outcomes. These results refine understanding of how the lung volume in apnoea could interact with autonomic reflexes, including cardiovascular adjustments mediated by the diving reflex and with nociceptive modulation through baroreceptor and chemoreceptor pathways. From an integrative physiological perspective, these findings highlight the importance of characterising the conditions under which respiratory‐induced autonomic stressors may or may not translate into measurable changes in nociceptive processing. Delineating these mechanistic boundaries will inform future research on the integrative physiology of breath‐hold interventions, clarify the conditions under which nociceptive modulation occurs, and support the design of experimental protocols probing cardiovascular, respiratory and pain control mechanisms in exercise physiology.

### 4.1 Conclusions

In this pilot trial, no meaningful between‐group differences in PPTs were observed following static low‐lung‐volume apnoea in healthy participants. Observed effect sizes were small. Confidence intervals were narrow for the tibialis anterior, suggesting a likely absence of a meaningful effect, but were wider for the thumb and C7, limiting interpretability. Although the AG reported slightly greater discomfort, the protocol was not associated with physiological risks or clinically relevant adverse events. Further studies should examine alternative apnoea configurations, including different durations, the addition of exercise and evaluation in pain populations, to clarify whether other approaches may produce meaningful hypoalgesic effects.

## AUTHOR CONTRIBUTIONS

Project administration, resources, and supervision were performed by José Fierro‐Marrero and Francisco De Asís‐Fernández; conceptualization and methodology by Cristian Mendoza‐Arranz, Álvaro Reina‐Varona, José Fierro‐Marrero, and Francisco De Asís‐Fernández; data collection by Cristian Mendoza‐Arranz, Guillermo Miravet‐Mingo and José Vicente León‐Hernández; writing, original draft by Cristian Mendoza‐Arranz; data analysis by Álvaro Reina‐Varona and José Fierro‐Marrero; writing, review and editing by Álvaro Reina‐Varona, José Fierro‐Marrero, José Vicente León‐Hernández, José Vicente León‐Hernández and Francisco De Asís‐Fernández. All authors have read and approved the final version of this manuscript and agree to be accountable for all aspects of the work in ensuring that questions related to the accuracy or integrity of any part of the work are appropriately investigated and resolved. All persons designated as authors qualify for authorship, and all those who qualify for authorship are listed.

## CONFLICT OF INTEREST

None of the authors and co‐authors have any conflict of interest to declare.

## GENERATIVE AI STATEMENT

During manuscript preparation, generative AI tools (ChatGPT, OpenAI, GPT‐5.3‐mini model) were used solely to support language editing and grammatical refinement. The authors reviewed and revised all content and take full responsibility for the accuracy and integrity of the manuscript. No AI tools were used for data collection, statistical analysis or generation of results.

## Supporting information



AppendixS1

MethodsS1

ResultsS1

ResultsS2

Supp Data Base

## Data Availability

The anonymized dataset and all supplementary materials supporting the findings of this study are publicly available in Zenodo at https://doi.org/10.5281/zenodo.21701010, distributed under the Creative Commons Attribution 4.0 International (CC BY 4.0) license.
