# Supplementary material for: Physiological and autonomic responses to intermittent low‐lung‐volume apnoea and pain sensitivity: A randomized controlled pilot trial
Source: Exp Physiol. 2026 Sep 4;111(10):4928–41. doi: 10.1113/EP093960 (PMC13544601; doi:10.1113/EP093960)
Supplement: Supplementary file 1 [file EPH-111-4928-s002.docx]

**ResultsS1**

[**1. Sociodemographic and baseline data – Descriptive figures**	2](#_Toc226403629)

[**2. Graphics and model assumptions of Thumb, Tibialis and C7 PPTs**	18](#_Toc226403630)

[**3. Descriptives statistics, graphics and model assumptions of SpO_2_**	21](#_Toc226403631)

[**4. Descriptive statistics, graphics and model assumptions of %HR_max_.**	27](#_Toc226403632)

[**5. Model assumptions and results of intrasession SBP and DBP**	29](#_Toc226403633)

[**6. Model assumptions of seated SBP, DBP and Borg CR-10**	31](#_Toc226403634)

**1. Sociodemographic and baseline data – Descriptive figures**

**Supplementary figure 1.1 Density plot, Scatter plot and Q-Q plot for the variable age in both groups.**

**Supplementary figure 1.2 Density plot, Scatter plot and Q-Q plot for the variable BMI in both groups.**

**Supplementary figure 1.3 Density plot, Scatter plot and Q-Q plot for the variable Theoretical HR_max_ in both groups.**

**Supplementary figure 1.4 Density plot, Scatter plot and Q-Q plot for the variable Rest %HR_max_ in both groups.**

**Supplementary figure 1.5 Density plot, Scatter plot and Q-Q plot for the variable SpO_2_ in both groups.**

**Supplementary figure 1.6 Density plot, Scatter plot and Q-Q plot for the variable SBP seated pre in both groups.**

**Supplementary figure 1.7 Density plot, Scatter plot and Q-Q plot for the variable DBP seated pre in both groups.**

**Supplementary figure 1.8 Density plot, Scatter plot and Q-Q plot for the variable GPAQ in both groups.**

**Supplementary figure 1.9 Density plot, Scatter plot and Q-Q plot for the variable PSQI in both groups.**

**Supplementary figure 1.10 Density plot, Scatter plot and Q-Q plot for the variable PSS in both groups.**

**Supplementary figure 1.11 Density plot, Scatter plot and Q-Q plot for the variable PPT thumb pre in both groups.**

**Supplementary figure 1.12 Density plot, Scatter plot and Q-Q plot for the variable PPT tibialis pre in both groups.**

**Supplementary figure 1.13 Density plot, Scatter plot and Q-Q plot for the variable PPT C7 pre in both groups.**

**Supplementary figure 1.14 Density plot, Scatter plot and Q-Q plot for the variable SBP supine pre in both groups.**

**Supplementary figure 1.15 Density plot, Scatter plot and Q-Q plot for the variable DBP supine pre in both groups.**

**Supplementary figure 1.16 Bar charts for the variables sex and apnoeist in both groups.**

# **2. Graphics and model assumptions of Thumb, Tibialis and C7 PPTs**

**Supplementary figure 2.1 Residuals vs. Fitted values plot, Residuals vs. Basal Thumb PPT and Q-Q plot in both groups.**

**Supplementary figure 2.2 Residuals vs. Fitted values plot, Residuals vs. Basal Tibialis PPT and Q-Q plot in both groups.**

**Supplementary figure 2.3 Residuals vs. Fitted values plot, Residuals vs. Basal C7 PPT and Q-Q plot in both groups.**

# **3. Descriptives statistics, graphics and model assumptions of SpO_2_**

**Supplementary figure 3.1 Observed SpO2 plot in both groups.**

**Supplementary figure 3.2 Observed SpO2 plot without cycle 1 in both groups.**

**Supplementary figure 3.3 SpO_2_ model assumptions: Residuals vs. Fitted values plot, Residuals vs. Basal SpO_2_, Residuals vs. Time and Q-Q plot in both groups.**

**Supplementary figure 3.4 SpO_2_ model assumptions without cycle 1 Residuals vs. Fitted values plot, Residuals vs. Basal SpO_2_, Residuals vs. Time and Q-Q plot in both groups.**

**Supplementary figure 3.5 Intrasession oxygen saturation (SpO_2_) without the first cycle predicted values. The figure indicates oxygen saturation values predicted by intrasession time (0-360 seconds), intrasession cycle (2-9) group, and baseline resting SpO_2_ values.**

**Oxygen saturation response without the first cycle**

After excluding the first cycle, a significant difference in SpO₂ remained at the reference time point (MD = 0.585%, SE = 0.294, t = 1.99, p = 0.0463), again with slightly higher values in the AG. No independent main effects of time (Time; t = −0.29, Time’: t = 0.26 and Time’’; t = −0.21, p > 0.05 for all) or cycle were observed. A significant Group × Time interaction (p < 0.0001 for all) persisted, indicating distinct temporal responses between groups, with the AG displaying an early decline, transient increase towards the end of the apnoea, and a later decrease in SpO₂, whereas the CG showed a more stable pattern across the same period. In contrast to the previous model, no significant Group × Cycle or Group × Cycle × Time interactions were identified, suggesting comparable SpO₂ evolution across cycles when the first cycle was omitted (Supplementary figure 4.5). Detailed model outputs are available in Supplementary Material 2 – GLS Intrasession SpO₂ and GLS Intra no 1st cycle SpO₂.

# **4. Descriptive statistics, graphics and model assumptions of %HR_max_.**

**Supplementary figure 4.1 Observed %HR_max_ plot in both groups.**

**Supplementary figure 4.2 %HR_max_ model assumptions: Residuals vs. Fitted values plot, Residuals vs. %HR_max_, Residuals vs. Time and Q-Q plot in both groups.**

# **5. Model assumptions and results of intrasession SBP and DBP**

**Supplementary figure 5.1 SBP model assumptions: Residuals vs. Fitted values plot, Residuals vs. Basal SBP, Residuals vs. Time and Q-Q plot in both groups.**

**Supplementary figure 5.2 DBP model assumptions: Residuals vs. Fitted values plot, Residuals vs. Basal DBP, Residuals vs. Time and Q-Q plot in both groups.**

# **6. Model assumptions of seated SBP, DBP and Borg CR-10**

**Supplementary figure 6.1 Residuals vs. Fitted values plot, Residuals vs. Basal seated SBP and Q-Q plot in both groups.**

**Supplementary figure 6.2 Residuals vs. Fitted values plot, Residuals vs. Basal seated DBP and Q-Q plot in both groups.**

**Supplementary figure 6.3 Residuals vs. Fitted values plot and Q-Q plot in both groups.**

**Supplementary figure 6.4 Box plot in both groups**
