# Supplementary material for: Physiological and autonomic responses to intermittent low‐lung‐volume apnoea and pain sensitivity: A randomized controlled pilot trial
Source: Exp Physiol. 2026 Sep 4;111(10):4928–41. doi: 10.1113/EP093960 (PMC13544601; doi:10.1113/EP093960)
Supplement: Supplementary file 3 [file EPH-111-4928-s004.docx]

# **1. Statistical analysis**

Sociodemographic and baseline data were summarised using mean, SD, minimum, Q1, median, Q3 and maximum values by group for continuous variables and frequencies for categorical variables. This information was displayed with density plots, scatter plots (with jittered values and mean reference) and Q–Q plots. All the graphics for categorical variables were generated and presented in ResultsS1 section 1.

Intrasession values of SpO_2_, %HR_max_, SBP and DBP were plotted through time and clustered by groups, reporting: 1) mean ± SD; 2) min, Q1, median, Q3, and maximum values.

To analyze potential baseline differences between groups, the following model was applied:

Baseline Variable ~ ols(Group)

This approach evaluates group differences in baseline means using Ordinary Least Squares (OLS) regression.

Potential baseline differences between groups were analyzed using Ordinary Least Squares (OLS) regression. Assumptions of homoscedasticity, linearity and normality were visually assessed using residuals vs fitted values plots and Q-Q plots (Harrell, 2015). If both assumptions were met, OLS regression with bootstrap resampling and Percentile confidence intervals (CI) were used to estimate 95%CI and p–values (Efron & Tibshirani, 1994; Wright et al., 2011). The mean difference between groups was reported. If either assumption was violated, the Wilcoxon Sum Rank Test was used instead, in these cases, the W statistic, p–value, and median difference were reported.

For categorical variables Chi–square tests were used. When the expected cell counts in any cell were below 5, the Fisher’s exact test was applied, χ² and p–value were reported, additionally, for Fisher’s exact test, the odds ratio, 95% confidence interval, and exact p–value were also reported.

An OLS regression model was applied to analyze PPTs (C7, thumb, tibialis), Borg CR–10, seated SBP, and DBP. The model predicted post-intervention outcomes, including group as a predictor. Additionally, for PPTs, SBP, and DBP, baseline values of the respective variables were included as covariates, thus implementing an analysis of covariance (ANCOVA) framework to control baseline differences (Lai & Kelley, 2012; Vickers & Altman, 2001). The formula was as follows:

*Model* <- *ols(Post-intervention variable ~ Basal variable + Group, data = d, x = TRUE, y = TRUE)*

With the confirmation of homoscedasticity and normal distribution of residuals, OLS models were complemented with bootstrap resampling (2,000 resamples) to enhance the robustness and reliability of the estimates . Bootstrap procedures used pseudo–random sequences with fixed seeds to ensure replicability of results as follows:

*Model_boot* <- *bootcov(Model, B = 2000, seed = 8)*

A group contrast between Experimental and Control groups was conducted employing Percentile method for estimating 95%CI:

*contrast(Model_boot, list(Group = 'Experimental'), list(Group = 'Control'), usebootcoef = TRUE, boot.type = 'bca')*

The contrast results included the coefficient (Mean difference: AG-CG), adjusted baseline variable value, SE and Percentile 95% CIs. Interpretation of between-group differences was based on these results.

Finally, to analyse possible non-linear relationships between intrasession variables of interest and time, generalized least squares (GLS) regression models were fitted with a continuous autoregressive [CAR(1)] correlation structure to account for within–subject correlation over time (Harrell, 2015; Pinheiro & Bates, 2000; Pinheiro et al., 1999).

Each model included the Baseline value as a covariate, Group (CG vs AG), Time (0 to 39 seconds within cycle or 0 to 6 minutes), Intrasession cycle (1–9), and the: Group x Time, Group x Intrasession cycle, Intrasession cycle x Time and Group x Time x Intrasession cycle interactions, in the case of SpO₂ and %HRmax variables, or Group × Time interaction in the case of SBP and DBP variables. Baseline values and time were modelled with restricted cubic splines (four knots at the 0.05, 0.35, 0.65 and 0.95 quantiles for SpO_2_ and %HR_max_ variables and three knots at the 0.1, 0.5 and 0.9 quantiles for SPB and DBP). This decision was based on Harrell’s recommendations for small samples (<100), balancing flexibility and parsimony to minimize the risk of overfitting (Harrell, 2015). In this context, the results for factors analysed using restricted cubic splines (Time, Time × Group, Intrasession cycle x Time, Time × Group × Intrasession cycle, and Baseline) would derive from a linear coefficient (b) and the non–linear adjustment captured by the splines (b’ and b’’), allowing for the interpretation of both linear and nonlinear effects. The model was fitted employing the restricted maximum likelihood method as default. The formula used in the GLS model was:

SpO₂ and %HRmax variables:

*Gls(Intrasession variable ~ Baseline variable + Group * Intrasession cycle * rcs(Time, 4), correlation = corCAR1(form = ~ Time | ID/Intrasession cycle), data = Intrasession data, x = TRUE)*

SBP and DBP variables:

*Gls(Intrasession variable ~ rcs(Baseline variable,3) + Group * rcs(Time, 3), correlation = corCAR1(form = ~ Time | ID), data = Sys_intrasess_data, x = TRUE)*

For SpO₂ and %HRmax data, the Intrasession cycle variable represented the nine consecutive 40 seconds cycles (1–9), accounting for progression across the 360 seconds protocol. The Time variable represented the second-by-second trajectory within each cycle (0–39 s), repeated across cycles. Moreover, for the SpO_2_ analysis, the model was repeated excluding the first cycle due to aberrant behaviour during that cycle, which showed no change in saturation, likely attributable to a delay in the instrument's capture capacity. The Baseline variable corresponded to the resting pre-exercise value of the outcome of interest. For SBP and DBP, *Intrasession variable* referred to their values through every minute of intervention (from “0-1” until “5-6” minutes, accounting for a total of 6 measurements). *Time* accounted for those 6 repeated measurements, and *Baseline variable* included the supine basal resting values. Regression coefficients, SE, t-value and p-values would be reported. Finally, fitted values of the model would be represented graphically with its 95%CI.

The suitability of GLS and OLS models were analysed visually for assumptions, exploring homoscedasticity across the absolute residuals of the model, with the fitted values, the baseline variable values, and time (exploring the mean and SD of absolute residuals in the GLS model). In addition, normal distribution of residuals was explored with Q-Q plots. Model assumptions are presented in ResultsS1 sections 2 to 6. If homoscedasticity or normality of residuals was not present, the Wilcoxon Sum Rank Test was employed for testing potential differences in post–intervention values between groups:

*wilcox.test(Post-intervention variable* *~* *Group, data = d, exact = FALSE)*

All the statistical analyses were conducted employing R software v.4.1.1 (R Core Team, 2021) (R Core Team, 2021). R: A language and environment for statistical computing. R Foundation for Statistical Computing, Vienna, Austria. URL https://www.R-project.org/). The package *dplyr* v.1.1.4, was employed for data summarization (Wickham et al., 2023), along with *base* v.4.4.1 and *stats* v.4.4.1. For intra–session analysis *tidyr* v.1.3.1, was used (Wickham et al., 2024). The package *rms* v. 6.8-2 (Jr, 2025) was employed for OLS, GLS, and restricted cubic splines modelling. Finally, *nlme* v. 3.1-164 (Pinheiro et al., 2026) was used for the GLS correlation matrix and *ggplot2* v. 3.5.1 (Wickham et al., 2025) for generating plots.

**REFERENCES**

Efron, B., & Tibshirani, R. J. (1994). *An Introduction to the Bootstrap*. Chapman and Hall/CRC. https://doi.org/10.1201/9780429246593

Harrell, J. (2015). *Regression Modeling Strategies: With Applications to Linear Models, Logistic and Ordinal Regression, and Survival Analysis* (2nd ed.). Springer. https://doi.org/10.1007/978-3-319-19425-7

Jr, F. E. H. (2025, April 4). *rms: Regression Modeling Strategies*. https://cran.r-project.org/web/packages/rms/index.html

Lai, K., & Kelley, K. (2012). Accuracy in parameter estimation for ANCOVA and ANOVA contrasts: Sample size planning via narrow confidence intervals. *British Journal of Mathematical and Statistical Psychology*, *65*(2), 350–370. https://doi.org/10.1111/j.2044-8317.2011.02029.x

Pinheiro & Bates. (2000). *Mixed-Effects Models in S and S-PLUS*. Springer-Verlag. https://doi.org/10.1007/b98882

Pinheiro, J., Bates, D., & R Core Team. (1999). *nlme: Linear and Nonlinear Mixed Effects Models* (p. 3.1-168) [Dataset]. https://doi.org/10.32614/CRAN.package.nlme

R Core Team. (2021). *R: A Language and Environment for Statistical Computing*. R Foundation for Statistical Computing. https://www.R-project.org/

Pinheiro, J., Bates, D., DebRoy, S., Sarkar, D., Heisterkamp, S., Van Willigen, B., Ranke, J., & R Core Team. (2026). *nlme: Linear and Nonlinear Mixed Effects Models* (Version 3.1-169) [Computer software]. https://cran.r-project.org/web/packages/nlme/index.html

Vickers, A. J., & Altman, D. G. (2001). Statistics notes: Analysing controlled trials with baseline and follow up measurements. *BMJ*, *323*(7321), 1123–1124. https://doi.org/10.1136/bmj.323.7321.1123

Wickham, H., Chang, W., Henry, L., Pedersen, T. L., Takahashi, K., Wilke, C., Woo, K., Yutani, H., Dunnington, D., & Brand, T. van den. (2025, September 11). *ggplot2: Create Elegant Data Visualisations Using the Grammar of Graphics*. https://cran.r-project.org/web/packages/ggplot2/index.html

Wickham, H., François, R., Henry, L., Müller, K., & Vaughan, D. (2023, November 17). *dplyr: A Grammar of Data Manipulation*. https://cran.r-project.org/web/packages/dplyr/index.html

Wickham, H., Vaughan, D., Girlich, M., & Ushey, K. (2024, January 24). *tidyr: Tidy Messy Data*. https://cran.r-project.org/web/packages/tidyr/index.html

Wright, D. B., London, K., & Field, A. P. (2011). Using Bootstrap Estimation and the Plug-in Principle for Clinical Psychology Data. *Journal of Experimental Psychopathology*, *2*(2), 252–270. https://doi.org/10.5127/jep.013611
